# Clinical prediction score for prolonged length of hospital stay in aneurysmal subarachnoid hemorrhage

**DOI:** 10.1186/s12883-023-03279-3

**Published:** 2023-06-16

**Authors:** Bambang Tri Prasetyo, Ricky Gusanto Kurniawan, Beny Rilianto, Pratiwi Raissa Windiani, Kelvin Theandro Gotama, Sardiana Salam, Ita Muharram Sari, Eka Musridharta, Abrar Arham, Muhammad Kusdiansah, Lyna Soertidewi Kiemas, Mursyid Bustami

**Affiliations:** 1 Neurointervention Division, National Brain Center Hospital Prof. Dr. dr. Mahar Mardjono, Letjen Mt. Haryono Street, No. Kav. 11, East Jakarta, Indonesia; 2 Faculty of Medicine, Airlangga University, Surabaya, Indonesia; 3 Neurointensive Care, National Brain Center Hospital Prof. Dr. dr. Mahar Mardjono, Jakarta, Indonesia; 4 Department of Neurosurgery, National Brain Center Hospital Prof. Dr. dr. Mahar Mardjono, Jakarta, Indonesia; 5 Neuroscience Unit, National Brain Center Hospital Prof. Dr. dr. Mahar Mardjono, Jakarta, Indonesia

**Keywords:** Clinical score, Length of stay, Aneurysmal subarachnoid hemorrhage

## Abstract

**Background:**

Length of stay (LOS) is an important indicator of the optimization of health services and hospital financing efficiency in aneurysmal subarachnoid hemorrhage (aSAH) patients. The purpose of this study was to develop a scoring model to predict the LOS of patients with aSAH.

**Method:**

A clinical scoring was developed based on retrospectively collected data from the cerebral aneurysm registry of the National Brain Center Hospital, Jakarta, from January 2019 to June 2022. Multivariate logistic regression was used to determine the odds ratio for risk-adjusted prolonged LOS. LOS predictors were obtained based on the regression coefficients and converted into a point score model.

**Results:**

Of the 209 aSAH patients observed, 117 patients had prolonged LOS (> 14 days of hospital stay). A clinical score was developed with a range of 0–7 points. Four variables were chosen as predictors of prolonged LOS: the presence of high-grade aSAH (1 point), aneurysm treatment (endovascular coiling: 1 point; surgical clipping: 2 points), cardiovascular comorbidities (1 point), and hospital-acquired pneumonia (3 points). The score showed good discrimination with an area under the receiving operating characteristics curve (AUC) of 0.8183 (SE 0.0278) and a *p*-value for the Hosmer–Lemeshow (HL) goodness-of-fit of 0.9322.

**Conclusion:**

This simple clinical score reliably predicted prolonged LOS in aneurysmal subarachnoid hemorrhage cases and may aid clinicians in improving patient outcomes and decreasing healthcare costs.

**Supplementary Information:**

The online version contains supplementary material available at 10.1186/s12883-023-03279-3.

## Background

Aneurysmal subarachnoid hemorrhage (aSAH) is the most serious complication from a ruptured cerebral aneurysm, having high morbidity and mortality [1, 2]. The global incidence of aSAH is estimated at 9 per 100,000 person-years, with the highest incidence rates recorded in Japan (22.7/100,000 person-years), followed by Finland (19.7/100,000 person-years) and South and Central American countries (4.2 cases/100,000 person-years). One-third of patients with aSAH do not survive the first days to weeks after bleeding [2, 3]. In Indonesia, the prevalence of aSAH patients reaches 4.2%, with a mortality rate of up to 80%, a figure which continues to increase [4].

Treatment of aSAH is very complex, inevitably requiring more time and incurring more cost. At the same time, a longer length of stay (LOS) in the hospital increases patients’ risk of contracting nosocomial infections, resulting in higher complication rate or even mortality [2, 5]. Thus, early prediction of patients’ LOS and their prognosis are of considerable value in treatment decision-making, ensuring the provision of adequate care while maintaining cost efficiency, as well as yielding information for patients and their respective families. Indeed, models for predicting the outcome of aSAH have been developed, but a reliable prediction model for LOS among aSAH patients remains unavailable. Several factors have been implicated as predictors for LOS in cases of aSAH, namely patients’ baseline illness or demographics, clinical presentation, treatment received in hospital, as well as medical complications and comorbidities [2, 5, 6]. The purpose of this study was to identify factors which play a role in the LOS of aSAH patients and develop a scoring model to predict the LOS of aSAH cases in both intensive care units and wards.

## Methods

### Study design

We retrospectively reviewed all medical records of patients diagnosed with aSAH, who were admitted to the National Brain Center Hospital, East Jakarta, Indonesia, from January 2019 to June 2022. The exclusion criteria were death during hospitalization and incomplete records. We included the following 17 variables in our analysis as follows: age, gender, aSAH grade, hypertension, diabetes mellitus, dyslipidemia, personal history of smoking, family history of cerebrovascular disease, hospital-acquired pneumonia (HAP), cardiovascular comorbidities, hyponatremia, hypokalemia, mechanical ventilation, treatment of aneurysm, treatment of hydrocephalus, aneurysmal location and size. aSAH grade was based on the World Federation of Neurological Surgeons (WFNS) score, where aneurysms scoring 1–3 were low-grade, and those scoring 4–5 were high-grade [7, 8]. Cardiovascular comorbidities included atrial fibrillation, hypertensive heart disease, and congestive heart failure. Hydrocephalus treatment included lumbar drain, ventriculoperitoneal (VP) shunt, and external ventricular drain (EVD), while aneurysm treatment included conservative treatment, endovascular coiling, and surgical clipping. Angiographic features of interest were aneurysmal location and size.

We defined prolonged LOS as hospital stay longer than 14 days, which was based on several considerations. Extended LOS among aSAH patients is often associated with the occurrence of vasospasm, and previous studies demonstrated that patients with vasospasm following SAH had a LOS of more than 14 days. In addition, delayed neuroinflammatory state after aSAH reaches its peak 4–14 days after the onset of rupture [6, 9–14].

### Statistical analysis

All statistical analyses were performed using the statistical software STATA (version 16.0, StataCorp, Texas, USA). Nominal data were compared with the χ^2^ test. We applied logistic regression analysis to determine predictors of prolonged LOS in aSAH patients from variables with *p* < 0.25 on previous univariate analyses. From the results of the multivariable logistic regression analysis, variables with *p* < 0.05 were selected as independent predictors for prolonged LOS. The smallest regression coefficient on the variable was selected as a divisor to calculate points in the assessment model. We used ROC analysis as a valuable tool to evaluate our predictive score. Receiver operating characteristic (ROC) curves compare sensitivity versus specificity across a range of values for the ability to predict a dichotomous outcome. Areas under the receiving operating characteristic (ROC) curve (AUC) were used to evaluate discrimination. The area under the curve can have any value between 0 and 1, which is a good indicator of the goodness of the test. A perfect diagnostic test has an AUC of 1.0, whereas a non-discriminating test has an area of 0.5. The Hosmer–Lemeshow (HL) goodness-of-fit test and calibration curves assessed the applicability of the models.

## Results

**Fig. 1:**
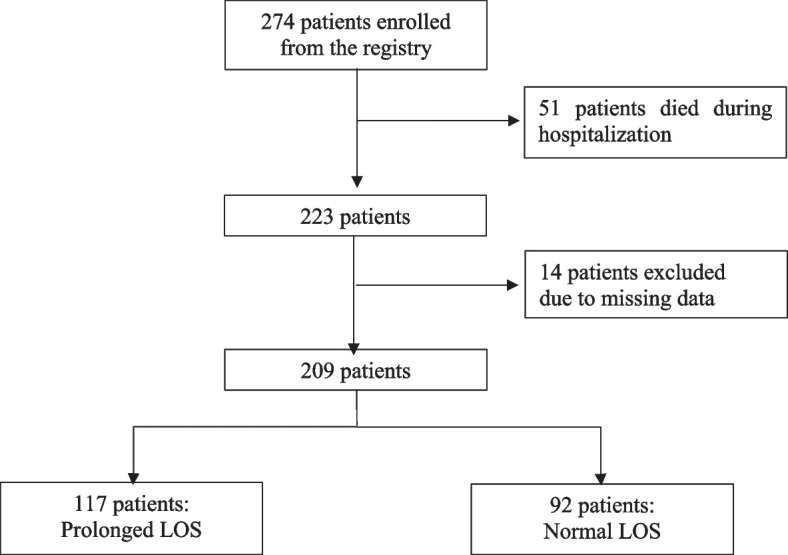
Subject flow diagram. LOS: length of stay

We enrolled 274 aSAH patients from the registry as our study subjects. Among those, 51 subjects died during hospitalization and 14 subjects had incomplete data. Therefore, we included 209 patients in the analysis (Fig. 1) Of these, 117 had prolonged LOS. The mean age of the patients was 53.39 years with a standard deviation of 11.30. Of the total cases, 139 were females and 70 were males, suggestive of a slight female preponderance with the ratio of 2:1. Demographic, vascular, clinical, and angiographic features are summarized on Table 1. Of the 17 variables, 13 variables exhibited a statistically significant association towards cases with prolonged LOS (*p* < 0.25): age of ≥ 60 years, female gender, high SAH grade, hypertension, diabetes mellitus, dyslipidemia, hyponatremia, hypokalemia, cardiovascular comorbidities, HAP, mechanical ventilation, treatment of aneurysm (coiling and clipping), and treatment of hydrocephalus (VP shunt and EVD) (Table 1).

**Table 1:** Demographic, vascular, clinical, and angiographic features of subjects

Variables	Prolonged LOS (> 14 days; *N* = 117)		Normal LOS (≤ 14 days; *N* = 92)		OR (CI 95%)	*p* value
	**Frequency**	**%**	**Frequency**	**%**		
**Demographic Characteristics**						
**Age (years)**						
< 40	8	6.84	9	9.78	Reference	
40–59	68	58.12	62	67.39	1.234 (0.448–3.397)	0.684
≥ 60	41	35.04	21	22.83	2.196 (0.740–6.519)	**0.156**
**Gender**						
Male	35	29.91	35	38.04	Reference	**0.216**
Female	82	70.09	57	61.96	1.439 (0.774–2.669)	
**Vascular Risk Factors**						
**Hypertension**						
Yes	96	82.05	69	75.00	1.524 (0.740–3.141)	**0.214**
No	21	17.95	23	25.00		
**Diabetes Mellitus**						
Yes	28	23.93	14	15.22	1.753 (0.820–3.864)	**0.118**
No	89	76.07	78	84.78		
**Dyslipidemia**						
Yes	49	41.88	31	33.70	1.418 (0.775–2.607)	**0.226**
No	68	58.12	61	66.30		
**Smoking**						
Yes	16	13.68	15	16.30	0.813 (0.352–1.889)	0.595
No	101	86.32	77	83.70		
**Family History of Cerebrovascular Disease**						
Yes	7	5.98	3	3.26	1.888 (0.415–11.603)	0.360
No	110	94.02	89	96.74		
**Clinical Risk Factors**						
**aSAH Grade (Based on WFNS Score)**						
Low (1–3)	74	63.25	81	88.04	4.279 (1.975–9.842)	**<0.001**
High (4–5)	43	36.75	11	11.96		
**Hyponatremia**						
Yes	72	61.54	38	41.30	2.274 (1.254–4.128)	**0.003**
No	45	38.46	54	58.70		
**Hypokalemia**						
Yes	73	62.39	39	42.39	2.254 (1.244–4.093)	**0.004**
No	44	37.61	53	57.61		
**Cardiovascular Comorbidities**						
Yes	26	22.22	8	8.70	3.0 (1.228–8.064)	**0.008**
No	91	77.78	84	91.30		
**Hospital-acquired Pneumonia**						
Yes	61	52.14	7	7.61	13.227 (5.452–36.336)	**<0.001**
No	56	47.86	85	92.39		
**Mechanical Ventilation**						
Yes	71	60.68	34	36.96	2.633 (1.445–4.812)	**0.007**
No	46	39.32	58	63.04		
**Treatment of Aneurysm**						
Conservative	18	15.38	32	34.78	Reference	
Coiling	34	29.06	36	39.13	1.679 (0.798–3.532)	**0.172**
Clipping	65	55.56	24	26.09	4.815 (2.289–10.126)	**<0.001**
**Treatment of Hydrocephalus**						
**Ventriculoperitoneal Shunt**						
Yes	96	82.05	69	75.00	4.018 (1.893–8.976)	**0.0001**
No	21	17.95	23	25.00		
**Lumbar Drain**						
Yes	28	23.93	14	15.22	1.593 (0.222–17.942)	0.592
No	89	76.07	78	84.78		
**External Ventricular Drain**						
Yes	49	41.88	31	33.70	4.206 (0.859–40.223)	**0.049**
No	68	58.12	61	66.30		
**Angiographic (Morphological) Features**						
**Location**						
Anterior Circulation	109	93.16	85	92.39	1.122 (0.332–3.697)	0.830
Posterior Circulation	8	6.84	7	7.61		
**Aneurysmal Dome Size**^a^** (*n* = 204)**						
< 5 mm	61	53.04	51	57.30	Reference	
5–10 mm	42	36.52	28	31.46	1.254 (0.684–2.298)	(0.464)
> 10 mm	12	10.43	10	11.24	1.003 (0.401–2.512)	(0.994)

*aSAH* aneurysmal subarachnoid hemorrhage, *CI* confidence interval, *LOS* length of stay, *OR* odds ratio, *WFNS* World Federation of Neurological Surgeons

^a^only available for saccular aneurysms

**Fig. 2:**
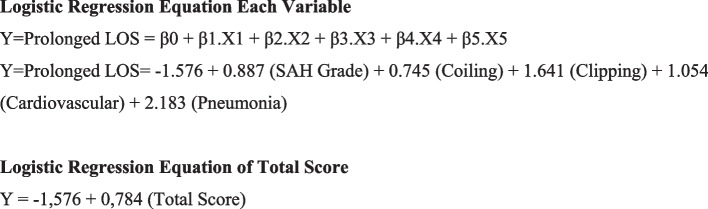
Logistic regression equation

We analyzed the 13 variables further using multivariable logistic regression to obtain a predictor model for LOS. There were 4 variables with *p* < 0.05, which were selected as independent predictors of prolonged LOS: (1) SAH grade — aOR (95% CI): 2.428 (1.012–5.824), *p* = 0.047; (2) aneurysm treatment — coiling aOR (95% CI): 2.106 (0.869–5.098), *p* = 0.009; clipping aOR (95% CI): 5.161 (2.137–12.463), *p* < 0.001; (3) cardiovascular comorbidities — aOR (95% CI): 2.869 (1.054–7.810), *p* = 0.039); and (4) hospital-acquired pneumonia — aOR (95% CI): 8.869 (3.517–22.364), *p* < 0.001 (Table 2). Regression coefficients on the selected variables were used to calculate points in the scoring assessment model (Fig. 2 and Table 3).

**Table 2:** Univariate and multivariate analysis of variables

Variables	Univariate Analysis		Multivariate Regression Model		
	**OR (95% Cl)**	***p*-value**	**β-Coefficient**	**aOR (95% Cl)**	***p*-value**
**Demographic Characteristics**					
**Age**					
< 40					
40–59	2.019 (0.488–8.347)	0.332			
> 60	2.446 (0.523–11.441)	0.256			
**Gender**					
Female	1.908 (0.778–4.679)	0.158			
**Vascular Risk Factors**					
Hypertension	1.232 (0.518–2.928)	0.636			
Diabetes Mellitus	0.953 (0.357–2.542)	0.923			
Dyslipidemia	0.779 (0.344–1.763)	0.549			
Smoking	1.337 (0.40–4.468)	0.637			
**Clinical Risk Factors**					
aSAH High Grade	2.384 (2.020–12.273)	0.082	**0.887**	**2.428 (1.012–5.824)**	**0.047**
Cardiovascular Comorbidities	3.203 (0.540–2.387)	0.036	**1.054**	**2.869 (1.054–7.810)**	**0.039**
Hospital-acquired Pneumonia	7.461 (0.390–4.795)	< 0.001	**2.183**	**8.869 (3.517–22.364)**	** < 0.001**
Hyponatremia	1.943 (0.298–18.598)	0.080			
Hypokalemia	1.219 (2.437–12.502)	0.606			
**Treatment of Aneurysm**					
Conservative					
Coiling	2.054 (0.804–5.249)	0.133	**0.745**	**2.106 (0.869–5.098)**	**0.009**
Clipping	5.392 (1.946–14.939)	0.001	**1.641**	**5.161 (2.137–12.463)**	** < 0.001**
**Airway Support**					
Mechanical Ventilation	1.038 (0.293–2.299)	0.930			
**Treatment of Hydrocephalus**					
Lumbar Drain	0.413 (0.468–3.641)	0.426			
VP Shunt	2.027 (0.789–5.211)	0.142			
EVD	3.424 (0.472–24.864)	0.224			
**Cons**	0.032 (0.004–0.212)	< 0.001	-1.576	0.207 (0.096–0.444)	< 0.001

*aSAH* aneurysmal subarachnoid hemorrhage, *CI* confidence interval, *EVD* external ventricular drain, *OR* odds ratio, *SAH* subarachnoid hemorrhage, *VP* ventriculoperitoneal shunt

**Table 3:** Scoring system for predicting prolonged LOS

Item	Category	Points
**aSAH Grade**	Low Grade	0
	High Grade	1
**Treatment of Aneurysm**	Conservative	0
	Coiling	1
	Clipping	2
**Cardiovascular Comorbidities**	No	0
	Yes	1
**Hospital-acquired Pneumonia**	No	0
	Yes	3
**Total Score**		**0–7**

*aSAH* aneurysmal subarachnoid hemorrhage, *LOS* length of stay

**Fig. 3:**
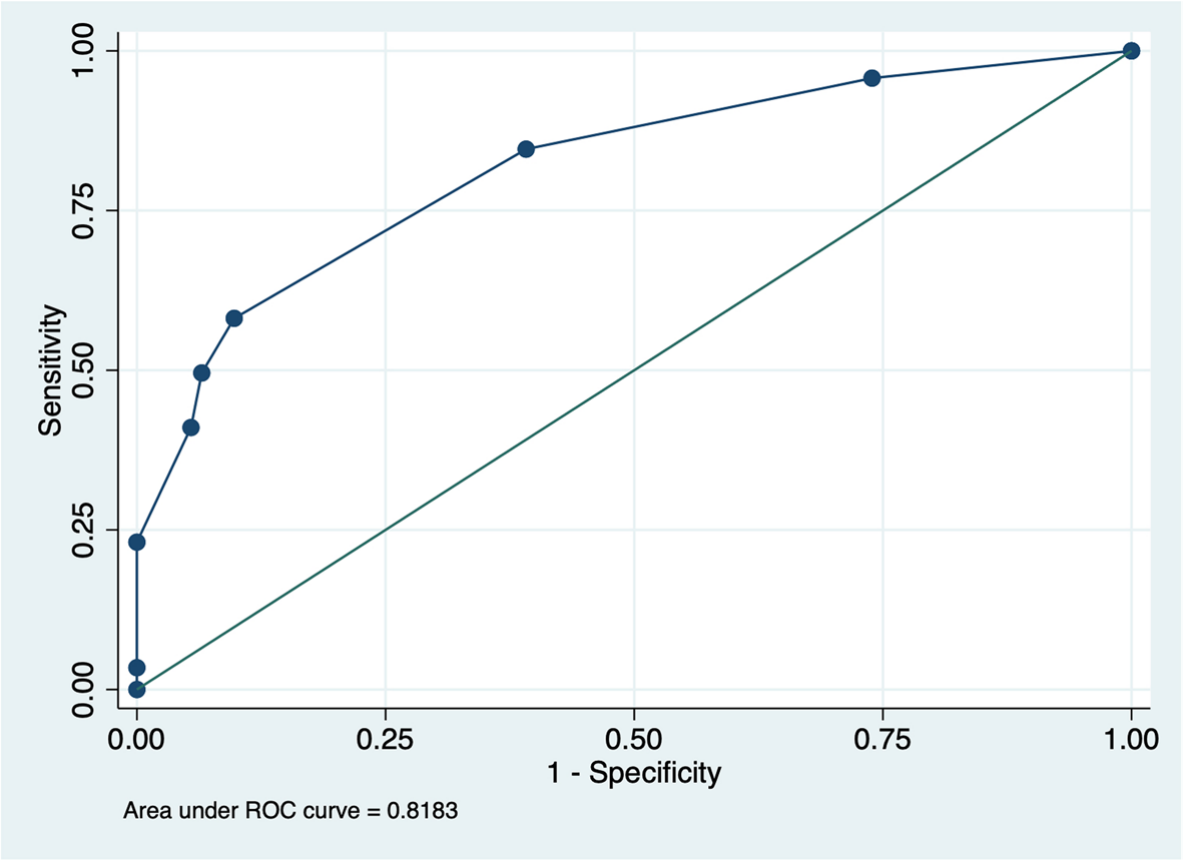
Receiver Operating Characteristics (ROC) curve with sensitivity and specificity of scoring

**Fig. 4:**
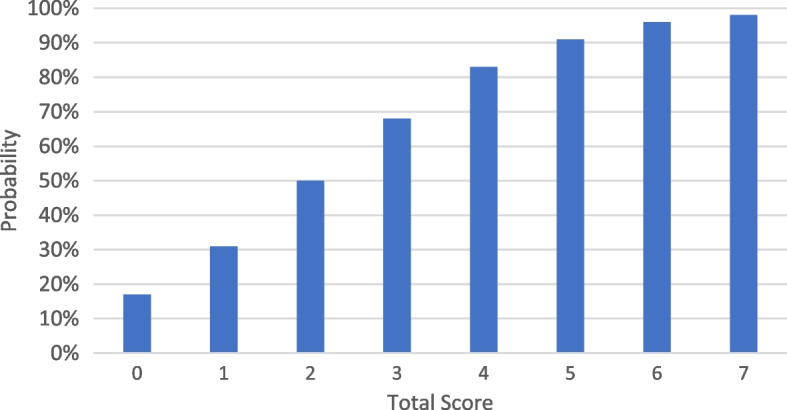
Probability of prolonged length of stay for each total score

The receiver operating characteristics (ROC) curve for sensitivity and specificity predictions performed by the new scale showed good accuracy with the area under the curve (AUC) of 0.8183 (SE 0.0278, 95% CI: 0.763–0.8727) in Fig. 3. Hosmer and Lemeshow's test showed a good model of discrimination (χ2 goodness-of-fit: 3.0342, p: 0.9322). Prognostic score was described with sensitivity, specificity as well as probability on Table 4 and Fig. 4.

**Table 4:** Prognostic score for prolonged length of stay

**Total Score**	**Sensitivity**	**Specificity**	**Y = -1.576 + ** **0.784 (Total Score)**	$\mathbf{p}=\frac{1}{1+{{\varvec{e}}}^{-({\varvec{y}})}}$	**%**
0	100	0	-1.576	0.171	**17**
1	96	26	-0.792	0.312	**31**
2	85	61	-0.008	0.498	**50**
3	58	90	0.776	0.684	**68**
4	50	93	1.560	0.826	**83**
5	41	95	2.344	0.912	**91**
6	23	100	3.128	0.958	**96**
7	3	100	3.912	0.980	**98**

## Discussion

The development of predictive models and assessments in the clinical approach towards aSAH patients has progressed in the recent decades. Unfortunately, its application remains suboptimal, owing to the underrepresentation of the study population, contentious degree of accuracy, and challenges in applying such experimental models in clinical settings [15]. Most of these scoring systems focused heavily on clinical outcome—but not LOS—and offered no consensus, utilizing a diverse range of variables and applying them to a heterogenous study population.

In our study, we predicted prolonged LOS using demographic characteristics, clinical information, location of the aneurysm, size of the aneurysm, and the treatment performed both surgically and conservatively. These variables were chosen because we believed that they were capable of reflecting the patient's clinical condition, which will describe their LOS in both the ICU and the ward. In this study, we also decided not to include other variables as they were missing in some medical records, which may potentially lead to bias. From those variables, four predictor factors displayed statistically significant correlation with prolonged LOS: aSAH grade, treatment of aneurysm, cardiovascular comorbidities, and HAP. We found that, utilizing these four predictors, our model predicted the LOS of our patients well. This can be seen statistically from the higher value of AUC (0.8183, SE 0.0278, 95% CI: 0.763–0.8727) in ROC analysis, as well as the specificity and sensitivity of each score (Table 4).

A notable scoring system is the SAFIRE (size of the aneurysm, age, Fisher grade, World Federation of Neurosurgical Societies after resuscitation) grading scale to predict functional outcome after aSAH. This score which independently predicted poor prognosis two months post-bleeding exhibited good discrimination (AUC 0.83, AUC 0.90 in temporal validation, and AUC 0.73 in external validation) [16]. Nevertheless, it is worth noting that the variables such as aneurysm size and Fisher's level can be susceptible to measurement errors, as they can be obtained through various imaging modalities and measurement techniques. In addition, the model did not include aSAH complications, even though they could significantly alter course of disease and affect the results.

Risseleda et al*.* also developed a prognostic model for aSAH-related mortality based on the International Subarachnoid Aneurysm Trial (ISAT) cohort (AUC 0.73), yet said model exhibited subpar performance when Dijkland et al*.* applied it to their study cohort, yielding a lower predicted mortality than actual case mortality [17, 18]. Indeed, the usage of mortality as the sole defining outcome for predictive models lends inferior clinical utility, as demonstrated by Jaja et al*.*, whose systematic review found that models predicting functional outcomes tend to perform better than those predicting mortality [15]. Another score, the FRESH score, was designed to predict cognitive outcomes and one-year quality of life of SAH patients based on Hunt and Hess scores, APACHE-II physiological score at admission, age, and aneurysm rebleeding within 48 h after rupture (AUC 0.77) [19]. However, Hunt and Hess scores rely on subjective observation by examiners and APACHE-II score utilizes laboratory parameters not readily available to physicians.

Some studies predict the outcome of aSAH using findings from neurological examinations, namely the level of consciousness, which are more straightforward to conduct. Zeiler et al*.* assessed the prognosis of aSAH cases based on the Full Outline of Unresponsiveness (FOUR) score, with the assessed outcome being death, the dichotomous 1- and 6-months Glasgow Outcome Scale (GOS) and modified Rankin Scale (mRS) values. They reported that FOUR scores at admission and seven days following SAH exhibited significant association with mortality as well as GOS/mRS at one month and six months [20]. Notably, the scoring system introduced brainstem scoring—a sub-score not found in the Glasgow Coma Scale (GCS) or the WFNS system—yet the number of patients with severe deficits and high-grade SAH in this study was too few to adequately establish the association between abnormal brainstem sub-scores and outcome [21–23].

In our study, we measured level of consciousness of patients with GCS and, by extension, graded SAH severity with the WFNS scoring system. We believe that WFNS remains the most robust predictor of poor outcomes in patients, being obtainable during initial patient assessment on admission, and reflecting the degree of cerebral tissue damage well [18, 21]. In our center, investigations to assess vasospasm, such as TCD or DSA, are not routinely performed on all patients. In addition, the severity of vasospasm is generally proportional to the WFNS grading. We, therefore, believe that the WFNS grading system encompasses the assessment of vasospasm. Our findings agreed with this; aSAH grading based on WFNS was an independent predictor for prolonged LOS following multivariate analysis. That said, deciding the course of treatment solely on the basis of WFNS and other parameters reflecting clinical presentation may not be sufficient. The symptomatology of aSAH reflects the intricacy of multiple pathological processes, such as intracerebral hemorrhage, cerebral edema, and acute hydrocephalus, to name a few [15, 21]. Therefore, incorporating other independent prognostic factors is necessary to enable a more comprehensive assessment of real clinical settings and, consequently, improve the model’s prediction.aSAH is closely related to the risk of hydrocephalus [24, 25]. Previous studies have suggested that the usage of EVD to treat hydrocephalus was associated with an increase in ICU LOS, likely because EVD weaning cannot be initiated until vasospasm and cerebral ischemia have subsided [24, 26]. In our study, there was a significant difference in the LOS between patients treated with VP shunt and EVD, but after adjusting, hydrocephalus treatment was not a predictor of prolonged LOS.

Our study found that pneumonia was more prevalent among patients with prolonged LOS than those with normal LOS, and we demonstrated that HAP independently predicted prolonged LOS. Therefore, patients with high risk of HAP need to be identified early. This is in agreement with Alaraj et al., who found that presence of pneumonia, as well as respiratory failure, significantly predicted prolonged LOS, even after multivariate analysis [27]. Pneumonia is a common complication following the occurrence of a stroke, but the pathophysiology behind the higher risk of in-hospital pneumonia from hemorrhagic stroke, when compared to ischemic stroke, remains obscure; some experts implicate the more severe neurological deficits in hemorrhagic stroke as the culprit, although this remains to be verified [28].

Cardiovascular comorbidities also predispose aSAH patients to serious problems that can occur during the hospital stay [29, 30]. It is postulated that acute SAH sets off a chain of neuroendocrine and inflammatory reactions which affects myocardial and vascular tissue; increased activations of the hypothalamus, insula, and brainstem were seen in SAH, indicating sympathetic activity [31]. Cardiovascular events, in turn, alters the course of disease, increasing delayed cerebral ischemia and mortality among aSAH patients [32]. In addition, Urbaniak et al*.* reported that an increase in morbidity as well as LOS (22.6 vs 17.1 days, *p* = 0.01) among aSAH patients with cardiovascular disorders [30]. This is in line with our investigation; we found the presence of cardiovascular comorbidities independently predicted prolonged LOS of aSAH patients.

We found that both treatments of cerebral aneurysm, endovascular coiling (*p* < 0.01) and especially surgical clipping (*p* < 0.001), were independent predictors of prolonged LOS. This confirms the findings of Hoh et al., who reported that among both ruptured and unruptured aneurysm patients, surgical clipping was significantly associated with prolonged LOS as well as higher hospital costs (*p* < 0.0001) when compared to coiling, and that aSAH patients treated with surgical clipping were hospitalized for 1.2-times longer than those treated with endovascular coiling on average [33]. Zhang et al. also found that patients with unruptured aneurysms who underwent coiling had significantly shorter LOS than those who underwent clipping (standard mean difference: 0.69, *p* < 0.001) [34].

Our scoring system is convenient to utilize as it relies on initial assessments performed during patients’ admission to the hospital, namely history taking, physical examination, and preliminary neurological examination. Therefore, we believe our scoring system may assist clinicians in estimating the LOS of their patients, enabling them to establish a course of treatment which maintains cost efficiency without sacrificing adequacy. We also expect this score to be applicable in healthcare centers with limited facilities, especially in developing countries such as ours.

### Study limitations

Nevertheless, there are several limitations to our study. This was a retrospective study, with moderate sample size, and single‐center data acquisition. There is no single scoring that can be universally applied for a particular outcome in all clinical practices. We hope our study can serve as one of the preliminary studies on the scoring system of aneurysm and will inspire future studies to develop future scoring systems with more predictors.

## Conclusion

This is the first simple scoring system—consisting of aSAH grade, aneurysmal treatment options, cardiovascular comorbidities, and HAP—which predicts prolonged LOS in aSAH. Further studies with multicenter acquisition and larger sample sizes are needed to validate the efficacy of this score.

## Supplementary Information

**Additional file 1.**

## Data Availability

The dataset generated and analyzed during the period of this study is included in the article (see Additional file 1). Any further inquiries may be directed to the corresponding author.

## Abbreviations

APACHE: Acute Physiology and Chronic Health Evaluation
aSAH: Aneurysmal subarachnoid hemorrhage
aOR: Adjusted odds ratio
AUC: Area under the curve
CI: Confidence interval
EVD: External ventricular drain
FOUR: Full Outline of Unresponsiveness
GCS: Glasgow Coma Scale
GOS: Glasgow Outcome Scale
HAP: Hospital-acquired pneumonia
HL: Hosmer-Lemeshow test
ICU: Intensive care unit
ISAT: International Subarachnoid Aneurysm Trial
LOS: Length of stay
mRS: Modified Rankin Scale
OR: Odds ratio
ROC: Receiving operating characteristic curve
SAH: Subarachnoid hemorrhage
VP: Ventriculoperitoneal
WFNS: World Federation of Neurological Surgeons
